# Supplementary material for: Biological data questions the support of the self inhibition required for pattern generation in the half center model
Source: PLoS One. 2020 Sep 11;15(9):e0238586. doi: 10.1371/journal.pone.0238586 (PMC7485810; doi:10.1371/journal.pone.0238586)
Supplement: Attachment — Submitted filename: Rebuttal_Letter.pdf [file pone.0238586.s001.pdf]

Rebuttal Letter

This manuscript was submitted to Plos One before.

Identification number: PONE-D-19-32768 - [EMID:54790cf2ae98afbf]

As required we provide detailed answers to the reviewers and editors comments.

Summary

The editorial decision to reject our manuscript might be biased, because the previously assigned editor, Jian Jing, has published extensively in the CPG field. Our paper is critical of the CPG concept. Reviewer#1 attests that our manuscript is technically sound and the data supports the conclusions. Reviewer#2s main concern is that our results are not not novel or significant. This is however subjective, as Reviewer#1 states that our results are interesting. Furthermore as stated by Reviewer#2 and stated in Plos Ones Editorial and Peer Review Process Guide, perceived novelty is not a requirement for Plos One. Reviewer#2 specifically states that our investigation on the half center model is not relevant in the field of the CPG. This statement is objectively incorrect as recent reviews, even the ones cited by the reviewer in the comments, are based on models of CPG organization containing half center models. The reason why Reviewer#2 attests only partial technical soundness is most likely that we did no statistical analysis. That is not an issue however, because we did not collect experimental data but did a modeling study. Note that Reviewer#1 attest that the statistical analysis has been performed appropriately and rigorously.

Reviewer #1

> This study is a modelling study where the authors investigate the half-center model and the rhythm generation in absence of endogenous pacemaker properties of the neurons that make up each half center. The authors suggest that there need to be a “self-inhibitory” mechanism for the system to oscillate. The self-inhibition can either be from a cellular process, the short-term depression (STD) or from a feedback inhibition from an inhibitory module that is added to the half-center model. The authors test both of these self-inhibitory mechanisms in computational models.
>
> The authors also pinpoint a number of shortcomings with the status quo half-center model and make several interesting observations. They find that the frequency is critically dependent on the time constants, either the STD recovery or the synaptic recovery, which are both relatively fixed and therefore make the rhythm generation rigid and unlikely. The authors argue that both these models are not

> working well in terms of covering the frequency range of locomotion (1-10 Hz oscillation) and therefore these mechanisms in themselves are not sufficient to explain locomotion. They conclude perhaps a completely different mechanisms is required. The study is rigorous and sound and the observations are interesting.

We point out that these comments are very positive to the paper in contrast to the second reviewer. Hence the perceived 'interestingness' of our paper to individual readers is subjective.

> My main objection to this study is the part that relates to self-inhibition from inhibitory interneurons, fig. 3A. Each half center has a local inhibitory population which is receiving excitatory input from the center (H1 or H2) and then project recurrent inhibition back to the center. This is essentially what is called a "balanced network" or "balanced excitation and inhibition", since the activity of excitatory neurons dampened by feedback from inhibitory neurons. This phenomenon has a widespread description in the literature in both theory and experiment - and the authors seem entirely unaware of.

We included a reference to these kinds of models, specifically the ones cited by the reviewer below and briefly explained how our models differentiate themselves.

> In particular, balanced input was experimentally observed in the spinal cord in connection with rhythmic movements (see Berg et al Science 2007 "Balanced inhibition and excitation drives spike activity in spinal half-centers") and a model very similar to Fig. 3A based on experiments was already proposed (Petersen et al J. Neuroscience 2014, "Premotor Spinal Network with Balanced Excitation and Inhibition during Motor Patterns Has High Resilience to Structural Division").

The model proposed in the cited paper from 2004 is similar to one of ours, except for that it receives external inputs from a CPG, in contrast to our model that constitutes a CPG that is independent of such inputs. The distinction is important since a periodicity in the former case might well depend on peripheral sensory input and would hence not be a CPG in meaning of a CENTRALLY generated pattern.

> Similarly, a network model akin to figure 3A was also modeled and proposed for the respiratory rhythm-generation see (Harris KD, Dashevskiy T, Mendoza J, Garcia AJ, Ramirez J-M, Shea-Brown E: Different roles for inhibition in the rhythm-generating respiratory network. J Neurophysiol 2017, 118:2070- 2288).

This paper demonstrates one model of the pre-Bötzinger complex. It utilizes a network structure that is similar to ours. It has a fixed set of parameters unlike our model where we explore a parameter space. We explore what parameters

that fulfill the requirements for locomotion and which of these parameters are physiological. Furthermore the neuron model in the paper has a slow inactivating sodium current. The time constant on it is 10000ms. As outlined in our introduction our models are made under the assumption that such a current is not active in vivo in adult mammals, because available biological evidence speaks against it. Hence our model differentiates itself at least from the model in the paper cited by the reviewer.

> A good review on the topic, which discusses the need for negative feedback mechanisms (what the authors call "self-inhibition") is provided in Berg et al Current opinion in Physiology 2019 "When networks walk a fine line: balance of excitation and inhibition in spinal motor circuits".
>
> Other comments:
>
> The "mean-field model" provided in lines 103-105 is really a Wilson-Cohen framework, which was already proposed and analyzed for this type of neuronal populations, see Wilson and Cowan Biophysical journal 1972 "Excitatory and inhibitory interactions in localized populations of model neurons"
>
> Line 55
>
> "A likely reason is that neurons in vivo have a much higher background synaptic conductance, which effectively shunt out prominent activation of inherent conductances that may still be inherent in the membrane of the neuron at adult age. This phenomenon has been demonstrated for the 58 deep cerebellar nuclear neurons [29], which have prominent intrinsic conductances in 59 juvenile brain slices, but there intrinsic conductances are normally not activated in vivo."
>
> Yes. In fact it has been shown in the adult spinal cord that during rhythmic activity the intrinsic conductances are in fact shunted, see Berg et al Plos One 2008 "Intense Synaptic Activity Enhances Temporal Resolution in Spinal Motoneurons " The authors show that the shunting is primarily due to both massive synaptic input and the concurrence of inhibition of excitation. This shunting also eliminates short term adaptation (also called spike-frequency adaptation) compared with the in vitro NMDA activated pacemaker rhythm.

We have no further comments, because the above sections is supportive of our paper.

Reviewer #2:

> My main concern with this manuscript is that, although PLOS One does not require a statement of significance or novelty of the results,

> the authors make no positive claim of anything.

Even though positive statements are not required for Plos One, as the reviewer points out, we discuss possible alternative explanations for locomotion that do not require a CPG. These are positive claims. Moreover, for any researcher operating in such a complex structure as the CNS, one should be open to the possibility that conceptually simplified models that are mapped as an interpretation of functionality of a highly complex dynamical system, can eventually be found to be an insufficient description. Hence, identifying this possibility can sometimes be as important an advance as trying to identify new such simplified models. This is the main contribution of our paper.

> In fact, the opposite is true. They begin by showing (using a complex network) that reciprocal inhibitory networks cannot generate a locomotion-type CPG, or any CPG in fact. This is a strange way to start, since that result is well-known already. It was in fact, already proposed by G. Brown himself in 1911 (for ex, reviewed by Friesen (1994) Neuroscience & Biobehavioral Reviews. Volume 18, Issue 4, 1994, Pages 547-553). As Brown himself proposed, a mechanism to terminate or wind down the inhibition is required.

It is true that what we show with the mean field model has been pointed out before. We cite one paper which does exactly that. Our contribution here is the mathematical formulation which is, contrary to the statement of the reviewer, a simple and small set of differential equations. The advantage with the mathematical formulation is that it is formally correct and hence the implied conclusions are indisputable, in contrast to previous more open-ended speculations to this same end.

> Short-term synaptic depression (STD) has been used by many (e.g. Taylor, Cottrell & Kristan Jr. (2002) Neural Computation, Volume 14, Issue 3, p.561-581; Skinner, Kopell & Marder (1994) J Comp Neurosc, vol 1). Yet, the authors present the idea of using STD as if it were an original one, which I do not understand.

The models in these cited papers by the reviewer are similar to the one featured in our paper. But our work shows that it cannot work for all frequencies of locomotion, which is a finding that has not been reported before. Furthermore we discuss which parameters in our model are in a physiological range.

> Several other mechanisms have been proposed (e.g. escape, rebound), that are based on the activation of slow outward currents or hyperpolarization-activated inward currents (Skinner, Kopell & Marder, 1994), which the authors ignore. Instead they argue at length in the introduction that a persistent inward currents could provide the self-inhibitory mechanism. Persistent inward currents have been shown to be the pacemaker currents in a number of systems, but I do not see how such a current can provide a

self-inhibition since they are, as their name indicates, inward and persistent.

How a persistent inward currents could result in spinal neurons having intrinsic pacemaking is extensively described in the papers that we cite when we describe persistent inward currents. Currently the CPG field focuses on persistent inward currents for which experimental support exists in vitro. This is also outlined in the recent review by Grillner & El Manira, cited below by the reviewer. Regarding persistent outward currents as proposed in (Skinner, Kopell & Marder, 1994), we are not aware of any experimental support for this in the mammalian spinal cord.

> The authors attempt to make the point that a half-center oscillator would be a natural CPG for mammalian locomotion and then try, and fail, to show how two alternative self-terminating mechanisms for each half's winding down of activity might work. This is surprising given that the research in the field does not support the idea of half-centers at all for either rhythm or pattern generation (Grillner & El Manira, 2019 Physiological Reviews). A different thing is the existence of reciprocal inhibition (which half-center oscillators require but are not sufficient to form a CPG). In any case, the field of mammalian locomotion does not currently accept that half-center oscillators are the mechanism to generate locomotion, and the authors' results do not support such a mechanism. To me, beyond the lack of novelty, which the journal does not require, this does not seem to me a consistent or interesting argument.

Figure 13, 14, 15 and 18 in the paper cited above show models of CPG organization which contain half center circuits. Hence our investigation into half center circuits is on topic.

> The point that the authors do make that I believe merits consideration is the range of locomotion frequencies observed in vivo (I presume in mice), which a single mechanism does not appear to be capable of generating. The authors, in fact, show that neither STD nor the alternative model that they present (self-inhibition via inhibitory interneurons) can generate such range. So, we are left none the wiser.

We discuss alternative explanations of locomotion which do not require CPGs.

> Minor comments. 1) There is no statistical analysis of the data.

This is true, because this is a modeling paper. We did not collect experimental data.

> 2) The authors claim that all the relevant data are presented in the manuscript. However, for

In the mail from the editor as well as in the attached to document in the mail, this comment is written like this, i.e. it appears to have been cut short. Since

this comment is incomplete we cannot respond to it.

> 3) Why does the model require such large pools of neurons for each element of the half-center and the inhibitory pools? You already make a large simplification by using IF models. Why not simplify the rest as well?

It would of course be possible to further reduce the models. We wanted to use spiking neurons, as many in the field of neurophysiology are used to this mode of operation of neuron models. Apart from that our models are simple because we use standard models provided by a commonly used neural simulator. Because we use spiking neurons the size of the neuron pool has to be set to some sufficiently large number. However the exact number does not influence the results.

> 4) On p. 5, beginning of paragraph 4. It is stated that "The general frequencies critically depend on the synaptic decay constant (Tau_in-syn) and the time constant of recovery from STD (tau_rec)". This is not what see in Fig. 2F. For different values of Tau_rec and of Tau_in-syn the values of 1/f appear to be quite constant (but variable). No clear dependency can be seen. Perhaps a more careful analysis and statistics and a figure showing this more clearly would help.

This comment contradicts itself. If 1/f would be constant the plotted points would form a plane parallel to the Ex_in tau_rec plane, which is clearly not the case. Something can not be constant and variable at the same time. The figure clearly shows that a non trivial dependency exists.

> 5) Figure 1 is trivial and completely unnecessary. At most, it could be an inset in Fig. 2.

We did not remove the figure because the existence of the figure does not influence the results.

> 6) The discussion could be simplified. It is much too long and mostly used to justify the negative results.

This is subjective, as we discuss many points that might be relevant for an interested reader.

> In fact, at the end of p. 8 and beginning of p. 9 the concept of passive mechanical walkers is invoked as a general mechanism of mammalian locomotion to make it consistent with their negative results.

We believe that an extensive discussion of the results is of greater value to an interested reader then only showing the results.

> This concept comes from the field of robotics and is not supported by neurophysiology of mammalian locomotion systems (at least the authors do not provide any evidence or references).

We do not present this hypothesis as a result but as speculative discussion.

> This concept is very similar to the original idea of Sherrington's of chains of reflexes, which I thought had been long discarded.

The CPG field has discarded this idea, but not the biological as well as the robotic locomotor field as witnessed by recent publications from several groups.

In total this reviewer has not made criticism that would question the validity of our methods or the results. His comments describe that he does not find our paper interesting. Of course this is a valid opinion but not a reason for rejection from Plos One.

Editor

> Your paper has been reviewed by two experts. Both reviewers raised a number of issues on your paper. In particular, Reviewer #2 was critical of your main claims or the lack thereof, although the reviewer did mention one topic that may be of interests.

Reviewer#2s main criticism is that he does not find the paper interesting. He made no comment that would question the validity of our methods and results. Indeed, as also stated by Reviewer#2 and stated in Plos Ones Editorial and Peer Review Process Guide, novelty is not a requirement for Plos One, since the judgment of that quality is subjective.

> Given these comments, it is likely that a required revision would result in a substantively different manuscript, thus, we cannot consider the manuscript in its current form. Instead, if you think you can address all the major points from both reviewers in a proper manner, we will consider a completely revised manuscript, which would be treated as a new submission.

The main points of criticism of both reviewers was addressed by including references to the papers suggested by the reviewers.

Finally we point out that the previously assigned editor himself has published extensively on the topic of CPGs, a concept that our results put into question.
