# Supplementary material for: Biological data questions the support of the self inhibition required for pattern generation in the half center model
Source: PLoS One. 2020 Sep 11;15(9):e0238586. doi: 10.1371/journal.pone.0238586 (PMC7485810; doi:10.1371/journal.pone.0238586)
Supplement: Attachment — Submitted filename: respone_to_reviewer.pdf [file pone.0238586.s002.pdf]

Response to the reviewer comments for submission PONE-D-20-07412

> Reviewer #1: Authors propose a notion that the half center model with self inhibition concept is the better model for CPG. This concept contributes to the development of robotics field such as walking machinery. However, from the point of view of electrophysiology, assumption that interneurons and/or inhibitory neurons is just inhibitory block (one parameter settings) seems to be too simple. Bursting could cause to increase the rise of intracellular Cl- concentrations. Eventually high concentrations of intracellular Cl- switch inhibitory GABAergic and glycinergic inputs to excitatory ones. Generally, frequency of firing of inhibitory neurons is much higher than that of excitatory neurons. In addition, intracellular Cl- homeostasis regulated by Cl- transporters is quite difference between juvenile and adult animals.

The process described by the reviewer could indeed provide an additional self inhibitory mechanism that we did not consider in the paper. The reviewer correctly points out that differences between juvenile and adult neuron physiology exist. In the adult a switch from inhibition to excitation does not seem to happen. Evidence for that exists from recordings in the spinal cord of adult cats, where the action of inhibitory neurons on motoneurons and interneurons was studied (Hultborn Jankowska Lindström 1971, Jankowska Roberts 1972). There high frequency stimulation evokes inhibitory postsynaptic potentials in the recorded neurons. However a switch from inhibition to excitation is not observed. Hence for the adult, which our manuscript is focused on, based on the available data, it is reasonable to assume that inhibition does not switch to excitation. We have updated the manuscript to to justify the usage of inhibition which can be reliably maintained.

For the juvenile, which our paper is not focused on, the situation is more confusing. It has been found that Gaba acts excitatory in juvenile neurons (Ben-Ari 2012). However counterevidence exists which shows that Gaba acts inhibitory in juvenile neurons (Kirmse et al. 2015, Valeeva et al. 2016).

During epilepsy GABA might have excitatory effects in the brain (Kaila et.al. 2014). If such effects are a potential cause for seizures, it is unlikely that they would be involved during locomotion.

References

Hultborn Jankowska Lindström 1971, Recurrent inhibition of interneurones monosynaptically activated from group Ia afferents https://doi.org/10.1113/jphysiol.1971.sp009488

Jankowska Roberts 1972, Synaptic actions of single interneurones mediating reciprocal Ia inhibition of motoneurones https://dx.doi.org/10.1113%2Fjphysiol.1972.sp009818

Ben-Ari 2012, The GABA excitatory/inhibitory Shift in Brain Maturation and Neurological Disorders https://doi.org/10.1177/1073858412438697

Kirmse et al. 2015, GABA depolarizes immature neurons and inhibits network activity in the neonatal neocortex in vivo https://doi.org/10.1038/ncomms8750

Valeeva et al. 2016, An Optogenetic Approach for Investigation of Excitatory and Inhibitory Network GABA Actions in Mice Expressing Channelrhodopsin-2 in GABAergic Neurons https://doi.org/10.1523/jneurosci.3482-15.2016

Kaila et.al. 2014 GABA actions and ionic plasticity in epilepsy https://doi.org/10.1016/j.conb.2013.11.004
